# Detection of quadratic phase coupling by cross-bicoherence and spectral Granger causality in bifrequencies interactions

**DOI:** 10.1038/s41598-024-59004-8

**Published:** 2024-04-12

**Authors:** Takeshi Abe, Yoshiyuki Asai, Alessandra Lintas, Alessandro E. P. Villa

**Affiliations:** 1 AI Systems Medicine Research and Training Center, Graduate School of Medicine and University Hospital, Yamaguchi University, Yamaguchi, 755-8505 Japan; 2 Department of Systems Bioinformatics, Graduate School of Medicine, Yamaguchi University, Yamaguchi, 755-8505 Japan; 3 HEC-LABEX, University of Lausanne, Quartier UNIL-Chamberonne, 1015 Lausanne, Switzerland; 4 Neuroheuristic Research Group & Complexity Sciences Research Group, University of Lausanne, Quartier UNIL-Chamberonne, 1015 Lausanne, Switzerland; 5 Division of Systems Medicine and Informatics, Research Institute of Cell Design Medical Science, Yamaguchi University, Yamaguchi, 755-8505 Japan

**Keywords:** Nonlinear interactions, Higher-order spectral analysis, Multivariate time series, EEG, Causal dependency, Neuroscience, Mathematics and computing

## Abstract

Quadratic Phase Coupling (QPC) serves as an essential statistical instrument for evaluating nonlinear synchronization within multivariate time series data, especially in signal processing and neuroscience fields. This study explores the precision of QPC detection using numerical estimates derived from cross-bicoherence and bivariate Granger causality within a straightforward, yet noisy, instantaneous multiplier model. It further assesses the impact of accidental statistically significant bifrequency interactions, introducing new metrics such as the ratio of bispectral quadratic phase coupling and the ratio of bivariate Granger causality quadratic phase coupling. Ratios nearing 1 signify a high degree of accuracy in detecting QPC. The coupling strength between interacting channels is identified as a key element that introduces nonlinearities, influencing the signal-to-noise ratio in the output channel. The model is tested across 59 experimental conditions of simulated recordings, with each condition evaluated against six coupling strength values, covering a wide range of carrier frequencies to examine a broad spectrum of scenarios. The findings demonstrate that the bispectral method outperforms bivariate Granger causality, particularly in identifying specific QPC under conditions of very weak couplings and in the presence of noise. The detection of specific QPC is crucial for neuroscience applications aimed at better understanding the temporal and spatial coordination between different brain regions.

## Introduction

The detection of nonlinear interrelations in multivariate time series is a crucial challenge in analyzing complex systems across diverse fields, including neuroscience, environmental science, economics, and engineering. Nonlinear signals can be characterized by higher-order statistics in both the time domain (such as higher-order cumulants) and the frequency domain (such as the Fourier transform of higher-order cumulants, leading to higher-order spectra)^1–4^. The inherent complexity of nonlinear dynamics means that interactions can be subtle, often obscured by noise or non-stationary signals. This subtlety requires careful methodological consideration to accurately distinguish causality from mere correlation in observed interrelations, thereby avoiding erroneous conclusions about the system’s underlying dynamics.

The analysis of time series by wavelet transforms provides insights into both time and frequency domains simultaneously^5–8^. This dual perspective is crucial for detecting nonlinear interactions that may vary over time. Alternatively, information-theoretic approaches quantify the flow of information between variables, capturing nonlinear dependencies through concepts such as mutual information, transfer entropy, and symbolic dynamics. Among these, mutual information (MI), especially when combined with Fourier transform surrogates, is particularly effective at distinguishing between linear and nonlinear interrelations and at detecting various forms of nonlinear interactions^9^. These interactions, potentially occurring at any order, offer insights into the underlying dynamics and interactions of systems—insights that are not accessible through linear analysis alone—including, but not limited to, phase synchronization, amplitude modulation, and more complex dynamics such as quadratic or higher-order phase coupling.

Phase coupling is a phenomenon where the phase (timing) of oscillations in one signal becomes correlated with the phase of oscillations in another signal. This implies that the two signals are not only correlated in their amplitudes but also in their timing. Quadratic Phase Coupling (QPC) goes a step further by capturing higher-order phase relationships between signals. It quantifies how the product of the instantaneous phases of two signals is related, indicating nonlinear phase interactions. The presence of QPC in a system, especially when compared to the detection of general nonlinearities is particularly noteworthy to detect the synchronization of biological rhythms or the modulation of signals in electronic and optical systems^10–12^. This is especially useful in fields like neuroscience, where understanding the phase relationships between neuronal signals detected by QPC might correlate with certain cognitive states, pathological conditions, or responses to stimuli and functional brain connectivity^13–20^.

Cross-bicoherence is a statistical measure used to assess phase coupling or nonlinear interactions between different frequency components in multivariate time series data. It is an extension of the bicoherence, which is a measure of the second-order spectral density of a signal. Cross-bicoherence specifically quantifies the degree to which the phases of two different signals at distinct frequencies are nonlinearly coupled or synchronized^21–23^. Establishing significance thresholds is essential for making reliable inferences about phase coupling in experimental data, particularly in applications such as neuroscience, where understanding brain connectivity and synchronization is of paramount importance. Several approaches have been carried out to develop statistical tests. Analytical methods assume specific statistical distributions for the cross-bicoherence values under the null hypothesis that its ground-truth value is zero^24,25^. Computational approaches include bootstrap resampling and permutation methods generating a large number of resampled datasets from the original data^26^ and Monte Carlo simulations generating surrogate data that mimic the statistical properties of the experimental data to create a null distribution^27^. By comparing the observed cross-bicoherence with the null distribution, statistical significance can be determined.

An alternative way to detect QPC is represented by bivariate Granger causality. The traditional Granger causality (GC) test is used to quantitative measuring whether one time series can predict another time series and to assess the magnitude of causal effects^28,29^. It can identify the direction of causality, thus determining which of the two time series leads in terms of causality^30^. Furthermore, GC goes beyond linear relationships, enabling the detection of nonlinear interactions between time series variables^31^. However, the conventional pairwise method for analyzing GC may not effectively differentiate between direct causal influences originating from one time series and indirect influences that operate through a third time series. This problem has been overcome by deriving a conditional GC in the frequency domain^32–34^. Such bivariate GC specifically determines the causal influence between two variables while accounting for the potential influence of other variables^35^. The corresponding statistics evaluation of significance include parametric tests^36,37^, nonparametric tests^38^ and various bootstrap testing approaches^34,39^. Nevertheless, estimation bias resulting from perturbations in observed time series data may generate potentially misleading statistically significant values, thus challenging true evidence of QPC even when considering the theoretical null distribution of the statistics.

The objective of this study is to identify specific QPC produced by the carrying frequencies of interacting channels within a basic, noisy instantaneous multiplier model. Our analysis revolves around examining the impact of a pair of interacting time series exhibiting varying degrees of coupling strength to a third time series. These time series represent stationary stochastic processes simulating experimental brain signals driven by independent oscillatory inputs, along with added noise. Our primary contribution lies in establishing a computational framework for the assessment of QPC detection. We utilize two analytical techniques: cross-bicoherence analysis and bivariate Granger causality. In both cases, we introduce an empirical Bayes method for the significance testing of the null hypothesis, specifically focusing on assessing whether the statistics are zero at given pairs of frequencies, known as bifrequencies. To evaluate the accuracy of QPC detection, we employ a false discovery rate inference methodology. This approach involves comparing the bifrequencies associated with significant statistics derived from the experimental time series to those expected from the phase coupling of the interacting channels’ carrying frequencies. Our findings indicate that the detection of specific QPC by cross-bicoherence analysis outperforms that by bivariate Granger causality, particularly in the presence of noise and when dealing with weak coupling among the interacting channels. We also discuss the potential applications of our results in the context of characterizing interaction networks, especially in the field of neuroscience.

## Results

### A three-channel model of bifrequencies interaction

We consider a simple model with three distinct channels labeled $X_1$, $X_2$, and $X_3$. Each channel receives input signals that are periodic, each one with its unique frequency denoted as $F_i$ (measured in Hz). The input signals are represented by three functions $I_1(t, \nu )$ (a triangular wave), $I_2(t, \nu )$ (a rectangular wave), and $I_3(t, \nu )$ (a cosine wave) as a function of continuous time *t* ($t \in {\mathbb {R}}$) and a sample index $\nu$. The use of triangular and rectangular waveforms is meant to endow our model with two advantageous properties. Firstly, the power distribution of these waveforms is more varied than that of pure sinusoids, thus being more closely mimicking the power-law spectrum commonly observed in nature. Secondly, the expected signal-to-noise ratio for each channel can be derived analytically (as detailed in the “Supplementary Material”), allowing for a more detailed analysis of the model’s performance in different conditions.

For each sample $\nu$, the initial phase parameter $\omega _i = \omega _i(\nu )$ of each signal $I_i$ is given a random shift. This shift follows a uniform distribution over the interval $[0, 2\pi )$, meaning the initial phase parameter can be any value between 0 and just under $2\pi$ radians, with all values within this range being equally probable. The periodic functions $I_i$ are defined as follows:

1 $$\begin{aligned} I_1(t, \nu )&= 2\, \text {asin}(\sin (2 \pi F_1 t + \omega _1(\nu ))); \end{aligned}$$

2 $$\begin{aligned} I_2(t, \nu )&= \left. {\left\{ \begin{array}{ll} 1 &{}\quad 0 \le (2 \pi F_2 t + \omega _2(\nu )) \bmod 2 \pi < \pi ;\\ -1 &{}\quad \text {otherwise} \end{array}\right. }\right. \end{aligned}$$

3 $$\begin{aligned} I_3(t, \nu )&= \cos (2 \pi F_3 t + \omega _3(\nu )) . \end{aligned}$$

**Figure 1:**
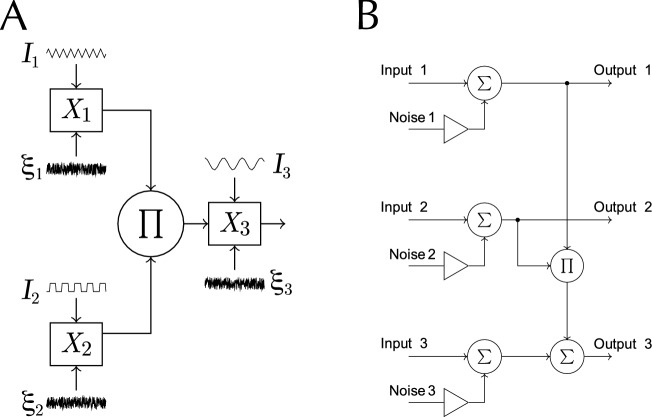
A three-channel model of bifrequencies interaction. (**A**) Schematic diagram of the model with input signal $I_i$, noise ${\xi }_i$, and output $X_i$ corresponding to channel *i*. (**B**) The corresponding block diagram.

An independent white noise is added to each channel ($\xi _1$, $\xi _2$, $\xi _3$), following a standard Gaussian distribution (mean $\mu =0$, standard deviation $\sigma =1$). In addition, channel $X_3$ receives an input from interacting channels $X_1$ and $X_2$ depending on a coupling strength $W_{(1,2)}$ (Fig. 1). The following equations define the three-channel model as a set of stochastic processes:

4 $$\begin{aligned} X_1(t, \nu )&= I_1(t, \nu ) + \xi _1(t, \nu ); \end{aligned}$$

5 $$\begin{aligned} X_2(t, \nu )&= I_2(t, \nu ) + \xi _2(t, \nu ); \end{aligned}$$

6 $$\begin{aligned} X_3(t, \nu )&= W_{(1,2)} X_1(t, \nu ) X_2 (t, \nu ) + I_3(t, \nu ) + \xi _3(t, \nu ). \end{aligned}$$

The detection of specific QPC was tested for distinct bifrequencies $(F_1, F_2)$ of channels $X_1$ and $X_2$ in the set of values $[({70/2\pi })$, $({80/2\pi })$, $({120/2\pi })$, $({150/2\pi })$, $({230/2\pi })$, $({50-10/2\pi })]$. We divided the parameter value of the frequency by constant $2\pi$ in order to cancel out the $2\pi$ in the argument of the trigonometric function in Eqs. (1–3). The carrying frequency $F_3$ of channel $X_3$ was chosen in the same set of frequency values, but distinct from $F_1$, $F_2$, and $F_1 + F_2$. The coupling strengths $W_{(1,2)}$ were chosen in the set [0.025, 0.050, 0.075, 0.150, 0.300, 0.750], ranging from weak to strong. Three time series of 64000 points were generated for an experimental condition defined by each set of parameters.

**Figure 2:**
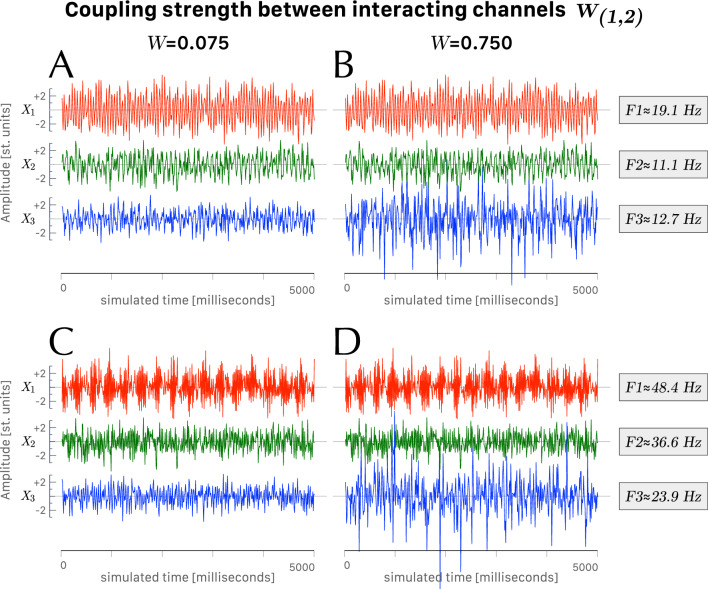
Four 5-second epochs of the three dimensional time series ($X_1$, $X_2$, $X_3$) for small coupling $W_{(1,2)}$ (panels **A**, **C**) and large coupling $W_{(1,2)}$ (panels **B**, **D**). (**A**, **B**) Experimental condition with carrying frequencies $F_1 = ({120/2\pi }) \approx 19.1$ Hz, $F_2 = ({70/2\pi }) \approx 11.1$ Hz, and $F_3 = ({80/2\pi }) \approx 12.7$ Hz. (**C**, **D**) Experimental condition with carrying frequencies $F_1 = ({50-10/2\pi }) \approx 48.4$ Hz, $F_2 = ({230/2\pi }) \approx 36.6$ Hz, and $F_3 = ({150/2\pi }) \approx 23.9$ Hz.

Each time series was created by merging 128 independent epochs, with each epoch corresponding to a 5-second interval sampled at 100 Hz. Each epoch contained an independent input signal phase parameters and white noise. Four 5-second epochs of the three-dimensional time series ($X_1$, $X_2$, $X_3$) are depicted in Fig. 2. It should be noted that the $X_1$ and $X_2$ time series are identical in panels Fig. 2A and C, as well as in panels Fig. 2B and D. The differences observed in the respective $X_3$ time series are solely attributed to a different coupling strength, $W_{(1,2)}$, between the interacting signals $X_1$ and $X_2$. Interestingly, a strong coupling strength leads to an overall amplification of the output signal $X_3$ in a nonlinear way, along with a predominant presence of frequency $F_3$ carried by channel 3. In total, 354 experimental conditions were examined (59 conditions for each value of coupling strength). For each condition we computed the $R_\mathrm{BQPC}$ (i.e., the ratio of bispectral quadratic phase coupling) and the $R_\mathrm{GQPC}$ (i.e., the ratio of bivariate Granger causality quadratic phase coupling).

### Quadratic phase coupling computed by cross-bicoherence and by Granger causality

**Figure 3:**
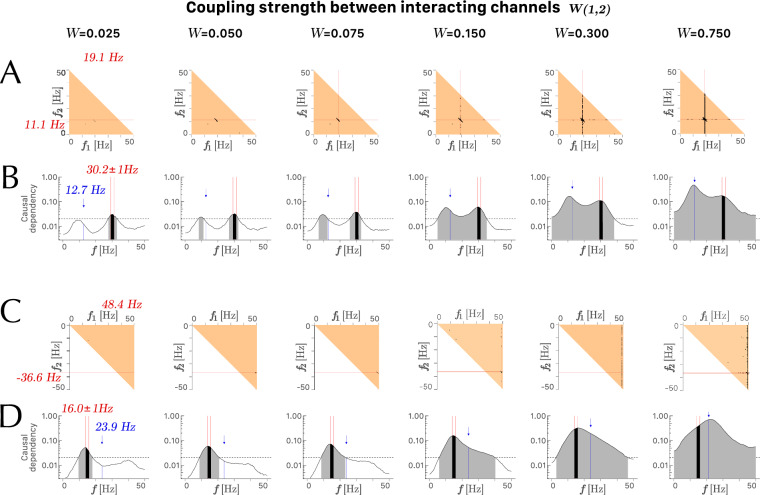
Examples of a three-channel model of bifrequencies interaction (Fig. 1) from channels $(X_1, X_2)$ to channel $X_3$ in the 0–50 Hz range with cross-channel coupling strength varying from weak to strong (from left to right). Panels in rows (**A**) and (**C**) show the cross-bicoherence significance maps with only those bifrequencies (black bins) characterized by cross-bicoherence values different from zero ($\overline{{b}^2_{ijk}}$, $q < .05$). Panels in rows (**B**) and (**D**) show the median unconditional bivariate Granger causality spectrum in a semi-logarithmic scale with a grey area corresponding to the integration over the curve of all significant values (with a threshold of significance represented by the dashed line) and a black area corresponding to the integration for the spectral interval of interest. (**A**, **B**) Analyses falling in sector $Q_{\textrm{I}}$ at bifrequencies $F_1 = ({120/2\pi })$ , $F_2 = ({70/2\pi })$ indicated by the red labels and $F_3 = ({80/2\pi })$ indicated by the blue label and arrow (i.e., $F_1 \approx 19.1$ Hz, $F_2 \approx 11.1$ Hz, $F_3 \approx 12.7$ Hz). (**C**, **D**) Analyses falling in sector $Q_{\textrm{II}}$ at bifrequencies $F_1= ({50-10/2\pi })$, $F_2 = ({230/2\pi })$ indicated by the red labels and $F_3 = ({150/2\pi })$ indicated by the blue label and arrow (i.e., $F_1 \approx 48.4$ Hz, $F_2 \approx 36.6$ Hz, $F_3 \approx 23.9$ Hz). In the plots, $f_1$ and $f_2$ correspond to the bifrequency coordinates of frequency parameters $(F_1, F_2)$.

We investigated how cross-bicoherence and bivariate GC were comparable in detecting quadratic phase coupling in two sectors of the cross-bicoherence domain for various coupling strengths(Fig. 3). Figure 3A,B illustrates the detection of QPC in sector $Q_{\textrm{I}}$ with $F_1 = ({120/2\pi })$, $F_2 = ({70/2\pi })$, and $F_3 = ({80/2\pi })$, and Fig. 3C,D illustrates the detection of QPC in sector $Q_{\textrm{II}}$ with $F_1 = ({50-10/2\pi })$, $F_2 = ({230/2\pi })$, and $F_3 = ({150/2\pi })$. Both methods show an increase in spurious values that affect the detection of specific QPC. In cross-bicoherence significance maps, these spurious values are represented by an increase in black bins along the axis corresponding to carrier frequencies $F_1$ and $F_2$, with an increase in coupling strength between the interacting channels $X_1$ and $X_2$ (Fig. 3A,C). In median unconditional bivariate GC spectrum, these spurious values are represented by an increase in overall significant causal dependency values and more specifically by a shift of the peak in this curve towards the value of the carrier frequency $F_3$ of channel $X_3$, represented by the blue labels and blue arrows (Fig. 3B,D).

**Figure 4:**
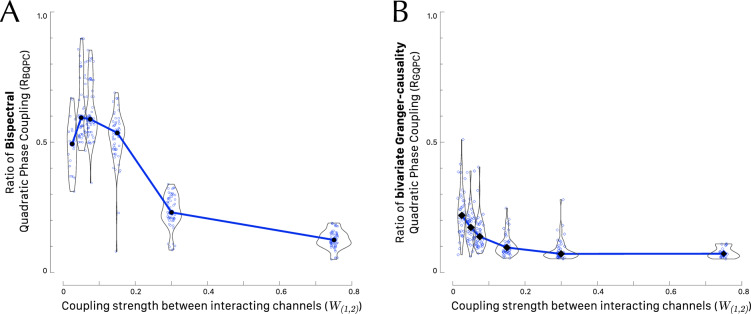
Effect of coupling strength between interacting channels $X_1$ and $X_2$ on the method chosen to detect quadratic phase coupling. The curves are centered on the medians of each group. The violin plots show the kernel probability density of the data at different values. (**A**) Ratio of bispectral quadratic phase coupling ($R_\mathrm{BQPC}$) as a function of the coupling strength $W_{(1,2)}$ between $X_1$ and $X_2$. (**B**) Ratio of bivariate Granger causality Quadratic Phase Coupling ($R_\mathrm{GQPC}$) as a function of the coupling strength. For graphical purpose the overlay of points has been limited by adding a random horizontal jitter equal to 0.05.

Building upon the preceding findings, the subsequent comparison of the specific QPC detection methods involves a two-way analysis of variance. This analysis incorporates the ratio of specific QPC as dependent variable, with three primary factors under consideration: method (consisting of 2 levels, “bispectral” and “bivariate GC”), coupling strength (comprising 5 levels, namely [0.025, 0.050, 0.075, 0.150, 0.300, 0.750]) and sector *Q* (consisting of 2 levels, $Q_{\textrm{I}}$ and $Q_{\textrm{II}}$). In this analysis, we have omitted the outlier values of the dependent variables, specifically, those with specific QPC ratios equal to zero or equal to 1. Both distributions of the indices show significant difference between variances across factor levels (Levene’s test $F(11, 292) = 6.62$, $p < .001$ and $F(11, 229) = 4.20$, $p < .001$ for $R_\mathrm{BQPC}$ and $R_\mathrm{GQPC}$, respectively). Therefore, the homogeneity of variances in the different factor levels cannot be assumed. The assumption of normality of the QPC ratios is not supported by the Shapiro-Wilk test ($p <.001$ for both $R_\mathrm{BQPC}$ and $R_\mathrm{GQPC}$). Then, our data did not meet the assumptions of the parametric ANOVA. As an alternative we run a three-way ANOVA on the ranks of QPC ratios. The method and the coupling strength $W_{(1,2)}$ are very significant factor variables ($F(1, 521)=37.249$, $p<.001$ and $F(5, 521)=44.753$, $p<.001$, respectively) as illustrated by Fig. 4 for both methods. The values of $R_\mathrm{BQPC}$ were larger than $R_\mathrm{GQPC}$ at any tested values of coupling strength (Wilcoxon tests, always $p<.001$ with a large effect size). Notice that the curve of $R_\mathrm{BQPC}$ is non-monotonic (Fig. 4A) with a median peak value of 0.497 occurring with $W_{(1,2)} = 0.050$, whereas the curve of $R_\mathrm{BGPC}$ is monotonically decreasing (Fig. 4B) with a median peak value of 0.119 at $W_{(1,2)} = 0.025$. It is also worth noting that in the three-way ANOVA the main factor sector *Q* is not significant ($F(1, 521)=1.906$, $p=.168$).

### Effect of coupling strength between interacting channels on signal-to-noise ratio

The signal-to-noise ratio (SNR) for any non-zero frequency of the input periodic signals $X_i$, is computed as

7 $$\begin{aligned} \textrm{SNR}_{X_i} = { P_{(X_i , \xi _i)} } / { P_{\xi _i} } , \end{aligned}$$

where , $P_{(X_i , \xi _i)}$ and $P_{\xi _i}$ correspond to the average (normalized) power for the signal and for the noise, respectively. In the three-channel model, the analytical solution for the computation of the signal-to-noise ratio yields (see Supplementary Material, section S1):

8 $$\begin{aligned} \textrm{SNR}_{X_1}&= \frac{\pi ^2}{3} ; \end{aligned}$$

9 $$\begin{aligned} \textrm{SNR}_{X_2}&= 1 ; \end{aligned}$$

10 $$\begin{aligned} \textrm{SNR}_{X_3}&= \left( \frac{2}{3} \pi ^2 + 2 \right) W_{(1,2)}^2 + \frac{1}{2}. \end{aligned}$$

In all 354 conditions examined, the SNRs of both experimental time series $X_1$ and $X_2$ consistently exceeded their respective expected values (Wilcoxon signed rank test, $p<.001$ for both $X_1$ and $X_2$). Conversely, in channel $X_3$, we observed that for 342 out of 354 conditions, the experimental SNR was consistently smaller than its corresponding expected value (Wilcoxon signed rank test, $p<.001$). This observed effect displayed a non-linear pattern, which was contingent on the coupling strength parameter $W_{(1,2)}$ (Kruskal–Wallis one-way ANOVA $\chi {^2}{_{(5)}} = 97.426$, $p<.001$).

**Figure 5:**
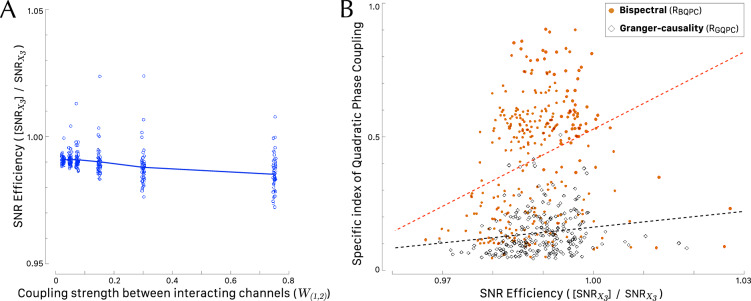
(**A**) Effect of coupling strength between interacting channels $X_1$ and $X_2$ on SNR Efficiency of channel $X_3$. The plot includes the result for all 354 conditions and for graphical purpose the overlay of points has been limited by adding random horizontal and vertical jitters of 0.005 and 0.002, respectively. (**B**) Effect of SNR Efficiency observed in channel $X_3$ on the novel specific indices of QPC associated with interacting channels $X_1$ and $X_2$. The values of $R_\mathrm{BQPC}$ are generally larger than $R_\mathrm{GQPC}$ at any value of the SNR Efficiency. The dashed lines correspond to trend lines computed according to Pearson’s correlation. For graphical purpose the overlay of points has been limited by adding a random horizontal and vertical jitter equal to 0.008.

The ratio $[{SNR}_{X_3}] / {SNR}_{X_3}$ of the experimental SNR divided by its analytical prediction (the expected value of the SNR) can be termed the SNR Efficiency. Higher values of SNR Efficiency (greater than 1) would indicate better-than-expected performance, whereas values less than 1 would suggest inefficiency. Figure  5A plots SNR Efficiency against the coupling strength and shows that SNR Efficiency is consistently underestimated (one-way Welch ANOVA with the coupling weights as factor and SNR Efficiency as dependent variable: $F(5,150.85) = 10.140$, $p < .001$). The underestimation of the numerical SNR of channel $X_3$ in comparison with its expected value is rather a positive finding; it indicates that our evaluation of $R_\mathrm{BQPC}$ or $R_\mathrm{GQPC}$ as statistics of QPC is favorably conservative. The observed decrease of SNR Efficiency with increasing coupling strength indicates that the discrepancy between the numerical value and its analytically expected counterpart widens as the coupling strength increases. It is noteworthy that the expected value, as derived analytically and detailed in the “Supplementary Material”, approaches an asymptotic limit. Conversely, the numerical value is influenced by the intrinsic amplitude of channel $X_3$, which itself is augmented with an increase in the coupling strength between the interacting channels $X_1$ and $X_2$.

The subsequent analysis focused on assessing how the SNR Efficiency in channel $X_3$ affected the detection of specific QPC by either bispectral analysis or bivariate Granger causality. Figure  5B illustrates that the values of $R_\mathrm{BQPC}$ consistently surpassed those of $R_\mathrm{GQPC}$, regardless of the SNR Efficiency (Wilcoxon signed rank test with continuity correction, $p<.001$ with a large effect size). Kendall’s rank correlation test revealed a statistically significant nonlinear dependence between the SNR Efficiency and both the novel specific indices we introduced, namely $R_\mathrm{BQPC}$ and $R_\mathrm{GQPC}$.

## Discussion

Quadratic Phase Coupling is essential for uncovering nonlinear interactions and complex relationships within multivariate time series data. Its application spans across multiple fields, including neuroscience, engineering, biology, and signal processing, where understanding nonlinear behaviors is crucial for making meaningful interpretations and improving modeling techniques. QPC can reveal how different brain regions or neurons interact nonlinearly during various cognitive tasks or in different brain states and thus can contribute for advancing our knowledge of brain functional connectivity, as shown in evoked potentials and EEG recordings^40–44^, local field potentials^45–47^, and magnetoencephalography^46,48^. The current study has introduced a straightforward interaction network model, exemplified by a noisy instantaneous multiplier showcasing characteristic QPC, specific to the carrying frequencies of the interacting channels. Our investigation revolved around examining bifrequency interactions originating from two channels $(X_1, X_2)$ to a third channel $(X_3)$ within the frequency range of 0–50 Hz range and varying the cross-channel coupling strength from weak to strong.

As the coupling strength between two interacting time series increases, we observe the emergence of spurious values that decrease the value of the new indices $R_\mathrm{BQPC}$ and $R_\mathrm{GQPC}$ (Fig. 4). This outcome might seem counterintuitive at first glance; one would typically expect the coupling parameter to solely enhance the significance of nonlinearity in the third series, $X_3$, rather than introducing anomalies. Indeed, as anticipated, we do observe increased cross-bicoherence and bivariate Granger causality across a broader band of bifrequencies for larger coupling strengths W(1,2), because these estimators quantify the QPC at any bifrequency (Fig. 3). However, stronger coupling also amplifies the likelihood that non-zero values of the estimators become significant, affecting not only to the signal’s component at the carrying frequency but also to the noise components ${\xi }_i$, present in the channels $X_1$ and $X_2$. These noise components exhibit a constant spectral density, except in the vicinity of the interacting bifrequencies $F_1$ and $F_2$. The characteristic of the new indices presented in this study is their dependency on the bispectral specificity of the coupling. Specifically, $R_\mathrm{BQPC}$ more effectively distinguishes coupling concentrated at a specific bifrequency and coupling distributed over a wider bispectral region than $R_\mathrm{GQPC}$.

In interacting networks, linear causal relationships revealed by Granger causality were initially applied in the field of econometrics^28^, and extended to study network interactions in biology, for example for determining the preferred direction of gene regulatory networks^49^ and metabolic networks^50,51^. However, the nonlinearity within interacting processes makes it difficult to apply parametric approaches for the characterization of recurring processes, such as threshold autoregressive moving average models that have been widely applied to financial time series analysis^52^. Concerns were raised that incorrect conceptual interpretation of causality approaches may lead to highly counterintuitive and potentially misleading results^53^. Observational noise can result in spurious causality detections, prompting the development of more rigorous estimations of Granger causality that have been successfully applied in experimental contexts in recent years^37,54–59^. Bivariate Granger causality, in particular, has proven highly effective in providing insights into how different signals influence each other’s phases^48,60^. This type of analysis is especially relevant in neuroscience, where the coupling of oscillatory activities at different frequencies between brain regions is crucial for the formation of functional networks. Other methods specifically sensitive to non-linear interactions (i.e., QPC) were developed and applied to EEG analysis^61,62^ However, designations of phase coupling and cross-frequency coupling may be misleading because effects of synchronization should be clearly distinguished from effects of signal transfer (propagation)^41,60,63^.

In situations where a signal contains harmonic components or intermodulation (where two or more frequencies mix to create new frequencies), higher-order spectral analyses prove valuable. They help distinguish these components and unveil their interrelationships. An alternative approach to Granger causality, aimed at gaining deeper insights into the characteristics of complex signals that go beyond what traditional power spectra and cross-spectra can reveal, encompasses methods such as the bispectrum and higher-order cumulants. When estimating parameters within the cross-bispectrum, it is assumed that the time series represent realizations of locally stationary processes, even when weak stationarity is present^3,21,25^. This approach relies on the fact that the bispectrum of a linear Gaussian process equals zero. Surrogate data can be used to test the null hypothesis that the original data were generated by a linear Gaussian process and QPC analysis is employed to detect and measure phase coupling that doesn’t adhere strictly to linear relationships^64^. To address potential issues related to mixing artifacts, robust solutions have also been suggested^65^. However, it’s important to note that the stationarity condition is a strong assumption. It implies that the probabilistic structure remains unchanged even when there is a significant time shift. This assumption can introduce bias when computing cross-bicoherence^66^. To tackle non-stationarity, wavelet analysis has been employed^7,67^. A specific method, the general harmonic wavelet transform-based bicoherence using phase randomization, was developed to measure the co-modulation of oscillations between different frequency bands at various sleep stages^68^. It’s noteworthy that the simultaneous multiplier examined in our current study represents a model of frequency mixers. In this model, channel $X_2$ plays the role of the local oscillator, assuming that the input of channel $X_1$ is akin to the input to a mixer. This model, by definition, is stationary. If we view the system generating these processes as a digital filter, then cross-bicoherence analysis reveals the filter’s properties. Consequently, our model provides an ideal framework for comparing the accuracy of specific QPC detection between cross-bispectrum and bivariate Granger causality.

The study of neural interactions and their implications for cognitive functions and neurological disorders underscores the importance of detecting specific patterns of QPC. Such detection is crucial for neuroscience applications aimed at better understanding the temporal and spatial coordination between different brain regions. In clinical neuroscience research, identifying aberrant QPC patterns, such as in the study of epilepsy, could help pinpoint the epileptogenic zones responsible for initiating and propagating seizure activity^13,44,69–71^. This information is critical for guiding surgical interventions or developing targeted neuromodulation therapies. Similarly, in Alzheimer’s disease, altered QPC patterns could serve as early biomarkers for disease progression or indicators of therapeutic intervention efficacy, thereby aiding in early diagnosis and the evaluation of treatments^45,72–74^. In the future, the detection of QPC could play a key role in neurorehabilitation, where understanding the reorganization of neural networks following injury or in response to therapy is paramount. Analyzing changes in QPC patterns in EEG or magnetoencephalography recordings before and after rehabilitation interventions could assess the neural basis of recovery and functional improvement, potentially leading to more personalized and effective rehabilitation strategies. Furthermore, by identifying specific QPC patterns from participants engaged in tasks requiring sustained attention or memory manipulation, the brain rhythms and dynamic networks activated during these cognitive processes could be elucidated.

In conclusion, cross-bicoherence is a valuable tool for detecting and quantifying phase coupling in multivariate time series data, especially in situations where nonlinear interactions are expected. We presented a simple model exemplifying how higher-order spectral analysis helps identify and mitigate interference, and enhance the detection of weak signals in noisy environments. QPC can uncover hidden patterns or relationships that might be missed by linear methods and an innovative computational approach was developed to evaluate the accuracy of QPC detection using different and complementary approaches. Our results revealed that cross-bicoherence, in comparison to a similar metric derived from bivariate Granger causality, offers several advantages. Notably, cross-bicoherence exhibits enhanced capability in detecting specific QPC resulting from the weak coupling of the interacting channels, demonstrating greater robustness against input noise.

## Methods

### Cross-bicoherence analysis

In this work, we consider a three-dimensional real vector *X* at time *t* as a particular multivariate time series or a realization of stochastic process that has polyspectra^1,3,75^,

$$\begin{aligned} X(t) = \begin{bmatrix} X_i(t) \\ X_j(t) \\ X_k(t) \end{bmatrix} \end{aligned}$$

where $X_i(t)$, $X_j(t)$, and $X_k(t)$ are three time series comparable to experimental brain signals. In particular, let us assume that *X*(*t*) is jointly stationary over the time period of observation and ${\tilde{X}}_i(f)$, ${\tilde{X}}_j(f)$, and ${\tilde{X}}_k(f)$ are the corresponding discrete Fourier transform at frequency *f* of each time series. After an appropriate preprocessing if necessary, we can assume that each time series is of mean $\mu =0$ and variance $\sigma ^2=1$ without loss of generality. For any two time series $X_i(t)$, $X_j(t)$, the cross-spectrum at frequency *f* is estimated by:

11 $$\begin{aligned} {\hat{C}}_{ij}(f) = {\widetilde{X}}_i(f) {\widetilde{X}}_j^*(f) \end{aligned}$$

where the superscript ($^*$) stands for the complex conjugate transpose. For a single time series $X_i(t)$, the *bispectrum* at two frequencies $f_1$ and $f_2$ is estimated by:

12 $$\begin{aligned} {\hat{B}}_i(f_1, f_2) = {\widetilde{X}}_i(f_1) {\widetilde{X}}_i(f_2){\widetilde{X}}_i^*(f_1 + f_2). \end{aligned}$$

For a three-dimensional time series *X*(*t*), the *cross-bispectrum* at frequencies $f_1$ for $X_i$ and $f_2$ for $X_j$ is estimated by the third joint moments of these processes:

13 $$\begin{aligned} {\hat{B}}_{ijk}(f_1, f_2) = {\widetilde{X}}_i(f_1) {\widetilde{X}}_j(f_2) {\widetilde{X}}^*_k(f_1 + f_2) \end{aligned}$$

The sample statistics of the magnitude-squared cross-bicoherence ${\hat{b}}^2_{ijk}$ simply referred to as *cross-bicoherence*, is a normalization of the cross-bispectrum as follows, where $\left\langle \cdot \right\rangle$ denotes the sample average (i.e., expected value of the sample):

14 $$\begin{aligned} {\hat{b}}^2_{ijk}(f_1, f_2) = \frac{ \left\langle \left|{{\hat{B}}_{ijk}(f_1, f_2)}\right|^2 \right\rangle }{ \left\langle \left|{{\tilde{X}}_i(f_1) {\tilde{X}}_j(f_2)}\right|^2 \right\rangle \left\langle \left|{{\tilde{X}}^*_k(f_1 + f_2)}\right|^2 \right\rangle }. \end{aligned}$$

It always holds that $0 \le {\hat{b}}^2_{ijk}(f_1, f_2) \le 1$ as the Cauchy–Schwarz inequality guarantees the upper bound. The values of *cross-bicoherence* can be used to interpret the interaction of $X_i$ and $X_j$ on $X_k$ (i.e., how well $X_k$ corresponds to $X_i$ and $X_j$) at each bifrequencies pair $(f_1, f_2)$. The estimation method used in the present work has been implemented and publicly available as an R package called $\texttt{rhosa}$^76^. Notice that the distribution of the estimated bicoherence converges to a noncentral $\chi ^{\,2}$distribution, if the null hypothesis holds that its genuine value is zero^77^. Furthermore, it can be approximated by a $\chi ^{\,2}$distribution^24^.

#### Estimation of a significance threshold based on the false discovery rate

We introduce a novel estimation of significant non-zero cross-bicoherence based on the false discovery rate (FDR) approach^78–80^. The estimation of significant non-zero cross-bicoherence is done on a large number of bins and a usual Type I Error equivalent to a confidence level of 95% ($p=0.05$) would imply that 5% of all tests result in false positives. While $p=0.05$ is acceptable for one test, it is not in case of a multiple testing problem. In FDR, *q*-values (or adjusted *p*-values) are determined for the tests that result in a discovery (i.e., a significant result), such that $q=0.05$ implies that 5% of significant tests (and not 5% of all tests) will result in false positives. Then, a cutoff $q=0.05$ will result in fewer false positives than a cutoff $p=0.05$.

In our novel procedure, we fit a two-class mixture distribution to the raw values of the cross-bicoherence for a given *q*-value. Assuming that *F* is the probability density of the cross-bicoherence in the trapezoidal domain of bifrequencies, we sought

15 $$\begin{aligned} F(x) = p_0 F_0(x) + [1 - p_0] F_{\textrm{A}}(x) \end{aligned}$$

where $F_0(x)$ is a $\chi ^{\,2}$distribution following the null hypothesis of zero cross-bicoherence; $F_{\textrm{A}}(x)$ is an alternative distribution; $p_0$ is the fraction of the statistic governed by the null hypothesis. The fitting is done by the mode matching method^81^ (which also provides a one-side test of the (positive) right-tail FDR against the empirical null distribution), with a fitting interval $p_0 = [0, 0.9]$ and bin width $\Delta = 0.001$. Hence, we denote $\overline{{b}^2_{ijk}}(f_1, f_2)$ the non-zero estimated cross-bicoherences significant to FDR with the assumption of a threshold $q=0.05$ of FDR for each significance testing.

#### Ratio of bispectral quadratic phase coupling ($R_\mathrm{BQPC}$)

**Figure 6:**
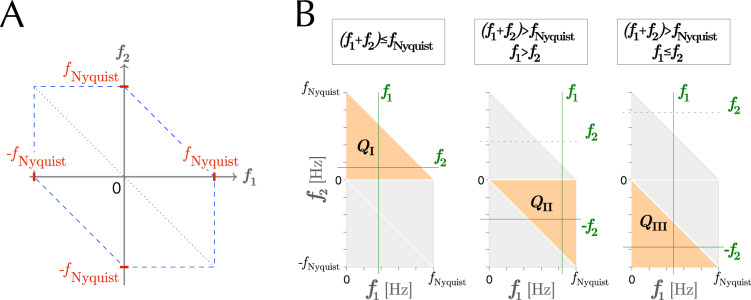
(**A**) The domain of cross-bicoherence corresponds to the distorted hexagon, surrounded by dashed lines. (**B**) The three sectors $Q_{\textrm{I}}$, $Q_{\textrm{II}}$, and $Q_{\textrm{III}}$ in the principal domain of cross-bicoherence of real-valued time series.

In a quadratic system, QPC occurs when the second-order nonlinearities of interacting $X_i(t)$ and $X_j(t)$ at bifrequencies $(f_1, f_2)$ (for $X_i$ and $X_j$, respectively) contribute to the power of $X_k(t)$. Given the symmetries in the distorted hexagonal cross-bicoherence domain^82–84^ (Fig. 6A), the QPC must be observed in the corresponding sector $Q(f_1, f_2)$ if the sum of the two interacting frequencies exceeds the Nyquist frequency (i.e., $f_1 + f_2 > f_{\textrm{Nyquist}}$). According to the three mutually exclusive conditions on bifrequencies $(f_1, f_2)$, the sectors $Q(f_1, f_2)$ are defined as follows (Fig. 6B):

$$\begin{aligned} Q(f_1, f_2) = {\left\{ \begin{array}{ll} Q_{\textrm{I}}&{}\quad \text {if}~(f_1 + f_2) \le f_{\textrm{Nyquist}}~; \\ Q_{\textrm{II}}&{}\quad \text {if}~(f_1 + f_2)> f_{\textrm{Nyquist}}~\text {and}~f_1> f_2~; \\ Q_{\textrm{III}}&{}\quad \text {if}~(f_1 + f_2) > f_{\textrm{Nyquist}}~\text {and}~f_1 \le f_2~. \end{array}\right. } \end{aligned}$$

The accuracy of experimental recordings is generally limited by the fact that measurements of brain activity is indirect. Small time delays and shifts in frequencies may be artificially produced by the recording techniques. Hence, an interaction occurring at bifrequencies $(f_1, f_2)$ is extended to a neighborhood of interest $G(f_1, f_2; d)$, such that $g_1 \in [f_1 - d,~f_1 + d]$ and $g_2 \in [f_2 - d,~f_2 + d]$, defined by the positive value *d* in frequency unit.

We introduce a novel index designed to quantify the quadratic phase coupling in the bispectral domain that is specifically associated with the carrying frequencies of the input signals from interacting channels. This index is distinct from a generic estimator, which can be influenced by significant non-zero values that are affected by noise components, ${\xi }_1$ and ${\xi }_2$, present in those channels. The ratio of bispectral quadratic phase coupling $R_\mathrm{BQPC}(f_1, f_2; d)$ is defined as the numerical integration of significant (i.e., $q \le 0.05$) cross-bicoherences in the neighborhood of interest divided by *all* significant cross-bicoherences in the relevant sector of bifrequencies (i.e., $(g_1, g_2) \in Q(f_1, f_2)$):

16 $$\begin{aligned} R_\mathrm{BQPC}(f_1, f_2; d) = \frac{\displaystyle \sum _{(g_1, g_2) \in G(f_1, f_2; d)} \overline{{b}^2_{ijk}}(g_1, g_2)}{\displaystyle \sum _{(g_1, g_2) \in Q(f_1, f_2)} \overline{{b}^2_{ijk}}(g_1, g_2)}. \end{aligned}$$

Notice that $G(f_1, f_2; d) \subset Q(f_1, f_2)$ and $R_\mathrm{BQPC}(f_1, f_2; d)$ is upper bounded to 1.

The frequency range (or bandwidth) of the spectrum is determined by the sampling frequency and, for a given sampling frequency, the spectral resolution $\Delta {f}$ (or resolution frequency) is determined by the number of points acquired in the recorded time series^85,86^. For example, an experiment acquiring 500 points at 100 Hz would yield an experimental frequency resolution $\Delta {f} = 100 / 500$ Hz, i.e. $\Delta {f} = 0.2$ Hz. Then, an actual frequency resolution $\Delta {f} = 0.25$ Hz in the bispectral domain of cross-bicoherence was determined as being the closest fractional value of power two.

**Figure 7:**
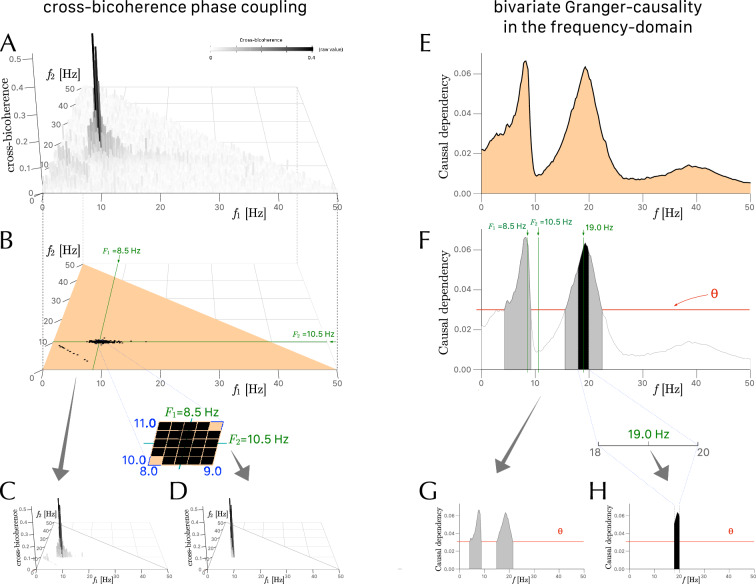
Three time series $X_1$, $X_2$, $X_3$ are recorded simultaneously (see text for more details). Estimation of the effect of quadratic phase coupling between $X_1, X_2$ for interacting bifrequencies ($F_1=8.5$ Hz, $F_2=10.5$ Hz) on $X_3$. The panels on the left side refer to the evaluation of the cross-bicoherence phase coupling and the panels on the right side refer to the evaluation of the bivariate Granger causality in the frequency-domain. (**A**) All cross-bicoherence values are computed in the sector $Q_{\textrm{I}}$. (**B**) The cross-bicoherence significance map, showing all bifrequencies (black bins) characterized by significant cross-bicoherence values. (**C**) The cross-bicoherence plot, showing all significant cross-bicoherence values whose sum is equal to 10.906 in this example. (**D**) The cross-bicoherence plot for the bifrequencies neighborhood of interest ($f_1 \in [8,9]$, $f_2 \in [10,11]$) (enlargement centered at a green cross mark), shows that the sum of these significant values is equal to 3.604, which yields $R_\mathrm{BQPC}= 0.330$ (i.e., 3.604/10.906). (**E**) For the same data, the median unconditional bivariate GC spectrum measures the strength of the causal dependency of the bivariate process $X_{1,2}$ on $X_3$ computed with the optimal VAR’s lag model, that is a VAR(7) model in this example. (**F**) A threshold value $\theta =0.0301$ is calculated to determine the significant values of Granger causality in the frequency domain. (**G**) The integration over the curve shows that the sum of all significant values is equal to 0.552. (**H**) For the spectral interval of interest ($f \in [18,20]$), the sum of all significant values is equal to 0.128, which yields $R_\mathrm{GQPC}= 0.233$ (i.e., 0.128/0.552).

This computation is illustrated by the analysis of a test data set formed by three artificially generated time series ($X_1$, $X_2$, $X_3$), recorded from 128 simulation runs of 5 seconds epochs sampled at 100 Hz. For time series formed by merging multiple epochs of the same duration, the Welch’s approach to frequency domain estimations is applied^87^ with an overlap of 50% for two consecutive data epochs. In this example, each time series is driven by a distinct periodic input ($F_1=8.5$ Hz, $F_2=10.5$ Hz, $F_3=9.5$ Hz) and the sources are interconnected. In particular, we compute $R_\mathrm{BQPC}(f_1, f_2; d)$ with $R_\mathrm{BQPC}(8.5, 10.5; 0.5)$ (i.e. a neighborhood of interest of 0.5 Hz around each frequency that participates to the interaction). In this case, the interaction occurs in the sector $Q_{\textrm{I}}$ (Fig. 7A). The estimation of a significance threshold based on FDR allows to plot a cross-bicoherence significance map (Fig. 7B) showing all bifrequencies characterized by cross-bicoherence values different from zero ($\overline{{b}^2_{ijk}}$). In this example, the sum of all significant cross-bicoherences in the sector $Q_{\textrm{I}}$ is equal to $\sum _{(g_1, g_2) \in Q(8.5, 10.5)} \overline{{b}^2_{ijk}}(g_1, g_2) = 10.906$ (Fig. 7C). We considered the bifrequencies neighborhood of interest $G(f_1 \in [8,9], f_2 \in [10,11])$. The sum of all significant cross-bicoherences in this neighborhood of interest is equal to $\sum _{(g_1, g_2) \in G(8.5, 10.5; 0.5)} \overline{{b}^2_{ijk}}(g_1, g_2) = 3.604$ (Fig. 7D). Hence, in this example $R_\mathrm{BQPC}= 3.604 / 10.906 = 0.330$.

#### Bivariate Granger causality in the frequency-domain

In the time-domain, the unconditional GC yields information on the strength of the causal dependency between two time series $X_i(t)$ and $X_j(t)$^30,33^. The GC of $X_j$ on $X_i$ compares a first model where the value of $X_i$ at time *t* is a linear weighted sum of its own past history (i.e., a linear vector autoregressive model, VAR)

17 $$\begin{aligned} X_i(t) = \sum _{m=1}^p a_m X_i(t-m) + \varepsilon _t \end{aligned}$$

with a second model where the value of $X_i$ at time *t* is a linear weighted sum of its own and of $X_j$ histories

18 $$\begin{aligned} X_i(t) = \sum _{m=1}^q b_m X_i(t-m) + \sum _{m=1}^q c_m X_j(t-m) + \eta _t \end{aligned}$$

where *p* and *q* are the lag orders of two models, and $a_m$, $b_m$, $c_m$ are real coefficients of the VARs, and $\eta _t$, $\varepsilon _t$ are terms corresponding to the residual errors. In other words, the GC of $X_j$ on $X_i$ measures if past values of $X_j$ yield information that predicts the next $X_i$ values better than solely the past $X_i$ history does.

In the frequency-domain, the Fourier representations of multivariate time series *X*(*t*) can be used to define GC with a spectral decomposition under some regularity conditions^88^. Let $\textrm{GCS}(X_i, X_j, f)$ denote the spectral GC of $X_i$ on $X_j$ at frequency *f*. For a bivariate stochastic process $X_{ij}(t)=[X_i(t),X_j(t)]$ (defined as the point-wise product of $X_i(t)$ and $X_j(t)$ at each time step *t*) in presence of a third time series $X_k(t)$, it is possible to extend the GC in the frequency-domain by defining the unconditional bivariate GC spectrum ${\textrm{BGCS}}_{ij\rightarrow k}(f) = {\textrm{GCS}}(X_i X_j, X_k, f)$. It measures the strength of the causal dependency of $X_{ij}$ on $X_k$.

In our practical implementation, the parameters of the vector autoregressive models (VAR(*l*) models) are computed using the $\texttt{vars}$ R package^89^. The VAR’s lag order *l* is selected according to the Schwarz Criterion (SC) (also called Bayesian information criterion, BIC)^90^ among other criteria because it offers the best probability of choosing the simplest possible model for large time series^91^. In principle, the smaller the SC value the better the fit of the model. However, very long time series are more likely to be overfitted and large lag orders could be incorrectly selected: the lowest SC value could be associated with lag orders without giving much more information about model fit. Hence, an heuristic approach is aimed to avoid overfitting: instead of choosing the VAR’s lag order *l* corresponding to the lowest SC, we select the lag order *l* corresponding to 95% of the lowest SC value with parameter $\mathtt {lag.max} = 50$.

The estimation of $\textrm{BGCS}_{ij\rightarrow k}$ follows the bootstrap method as implemented in $\texttt{grangers}$ R package^34^. The $\texttt{nboots}$ parameter (corresponding to the number of bootstrap series) is determined following the experimental spectral resolution $\Delta {f}$, i.e. $\texttt{nboots} = \sqrt{\Delta {f}} \times 1000$. We estimate the *q*-values of FDR from the set of *p*-values simultaneously calculated for all of Fourier frequencies in $(0, f_{\textrm{Nyquist}})$, using the R package called $\texttt{qvalue}$ and setting the parameter $\mathtt {pfdr = TRUE}$ for a more robust FDR estimation^92,93^. In case the variance of bootstrapped *p*-values is too small, the Benjamini-Hochberg procedure is used as a conservative alternative by setting the parameter $\mathtt {lambda = 0}$ for an estimation of the proportion of true null *p*-values. Following the FDR level of significance $q <.05$ for $\textrm{BGCS}_{ij\rightarrow k}$, we compute the value of causal dependency corresponding to the threshold $\theta$ which determines the significant values of the bivariate GC spectrum. With the Welch’s approach (with 50% overlap) for time series formed by merging multiple epochs of same duration, we compute the median $\textrm{BGCS}_{ij\rightarrow k}$ and the median value of threshold $\theta$ across all Welch’s data segments.

#### Ratio of bivariate Granger causality quadratic phase coupling ($R_\mathrm{GQPC}$)

Following the same approach determining $R_\mathrm{BQPC}$, we consider QPC occurring when a nonlinear interaction between $X_i(t)$ and $X_j(t)$ at bifrequencies $(f_1, f_2)$ produces a peak at frequency *f* in the unconditional bivariate GC spectrum ${\textrm{BGCS}}_{ij\rightarrow k}(f)$ computed for the optimal VAR’s lag order *l*. For an extension to experimental data, we consider the interaction at frequency *f* (determined by bifrequencies $(f_1, f_2)$) occurring within a spectral interval of interest $(f_1, f_2; d)$ (similar to the neighborhood of interest for the cross-bicoherence). Then, $f \in [(f_1 + f_2) - 2 d, (f_1 + f_2) + 2 d]$ if $(f_1 + f_2) \le f_{\textrm{Nyquist}}$ and $f \in [f_{\textrm{Nyquist}}- (f_1 + f_2) - 2 d, f_{\textrm{Nyquist}}- (f_1 + f_2) + 2 d]$ if $(f_1 + f_2) > f_{\textrm{Nyquist}}$ because of aliasing.

We introduce a novel index designed to quantify the quadratic phase coupling associated with Granger causality in a specific bivariate process. This index focuses on the carrying frequencies of the interacting channels, $X_1$ and $X_2$. The ratio of bivariate Granger causality quadratic phase coupling $R_\mathrm{GQPC}(f_1, f_2; d)$ is defined by the fraction of causal effects of the bivariate process $X_{ij}$ at a frequency *f* given $X_k(t)$:

19 $$\begin{aligned} R_\mathrm{GQPC}(f_1, f_2; d) = \frac{\displaystyle \sum _{f \in (f_1, f_2; d)} \overline{\textrm{BGCS}_{ij\rightarrow k}}(f)}{\displaystyle \sum _{f \in [0, f_{\textrm{Nyquist}}]} \overline{\textrm{BGCS}_{ij\rightarrow k}}(f)}. \end{aligned}$$

where $\overline{\textrm{BGCS}_{ij\rightarrow k}}(f)$ is the significantly non-zero value of the bivariate GC spectrum from $X_{ij}$ to $X_k$ at frequency *f*. Notice that $R_\mathrm{GQPC}(f_1, f_2; d)$ is upper bounded to 1.

This is illustrated in Fig. 7 using the same time series used to illustrate the computations for the cross-bicoherence. The median unconditional bivariate GC spectrum is calculated with a VAR(7) model, (i.e. VAR’s lag order $l=7$) and $\texttt{nboots} = 447$ (Fig. 7E). For this example, the median threshold value of 113 Welch’s data segments of 500 data points each is $\theta =0.0301035$ (Fig. 7F). The sum of all significant values of unconditional GC is equal to $\sum _{f \in (f_1, f_2; d)} \overline{\textrm{BGCS}_{1,2\rightarrow 3}}(f) = 0.552$ (Fig. 7G). The spectral interval of interest of the unconditional GC spectrum ($f \in [18,20]$) is determined by bifrequencies ($f_1=8.5, f_2=10.5$). The sum of all significant values of unconditional GC in this spectral interval is equal to $\sum _{f \in (f_1, f_2; d)} \overline{\textrm{BGCS}_{1,2\rightarrow 3}}(f) = 0.128$ (Fig. 7H). In this example $R_\mathrm{GQPC}= 0.128 / 0.552 = 0.233$.

## Supplementary Information

Supplementary Information.

## Data Availability

The time series that support the results of this study are available from the corresponding author upon reasonable request.

## Abbreviations

GC: Granger causality
QPC: Quadratic phase coupling
$R_\mathrm{BQPC}$: Ratio of bispectral quadratic phase coupling
$R_\mathrm{GQPC}$: Ratio of bivariate Granger causality quadratic phase coupling
