# Supplementary material for: Detection of quadratic phase coupling by cross-bicoherence and spectral Granger causality in bifrequencies interactions
Source: Sci Rep. 2024 Apr 12;14:8521. doi: 10.1038/s41598-024-59004-8 (PMC11372163; doi:10.1038/s41598-024-59004-8)
Supplement: Supplementary file 1 — Supplementary Information. [file 41598_2024_59004_MOESM1_ESM.pdf]

Supplementary material

Detection of quadratic phase coupling by cross-bicoherence and spectral Granger causality in bifrequencies interactions

Takeshi Abe[1,*], Yoshiyuki Asai[2], Alessandra Lintas[3], Alessandro E.P. Villa[4,*]

(1) AI Systems Medicine Research and Training Center, Graduate School of Medicine and University Hospital, Yamaguchi University, Yamaguchi, Japan

(2) Department of Systems Bioinformatics, Graduate School of Medicine, Yamaguchi University, Yamaguchi, Japan

(3) Neuroheuristic Research Group & HEC-LABEX, University of Lausanne, Quartier UNIL-Chamberonne, CH-1015 Lausanne, Switzerland

(4) Neuroheuristic Research Group & Complexity Sciences Research Group, University of Lausanne, Quartier UNIL-Chamberonne, CH-1015 Lausanne, Switzerland

*Corresponding authors:**
Takeshi Abe, Yamaguchi University
E-mail address: t.abe@yamaguchi-u.ac.jp

Alessandro E.P. Villa, University of Lausanne
E-mail address: alessandro.villa@unil.ch.

S1. Signal-to-noise ratio : SNR

For a signal $x_i(t)$, the average (normalized) power is given by (Grami, 2015):

$$P_{x_i} = \lim_{T\to\infty} \frac{1}{T} \int_{-T/2}^{T/2} |x_i(t)|^2 dt.$$

Let ν represent a sample i.e. realization of stochastic process $X_i (i = 1, 2, 3)$ generating time series of our three-channel model of bifrequencies interaction. For any independent white noise signal $\xi_i(t,\nu)$ following a standard Gaussian distribution (mean $\mu = 0$, standard deviation $\sigma = 1$), the average power is $P_{\xi_i} = 1$. For each channel $X_i(t,\nu)$, the power of the signal (without noise) is $P_{(X_i-\xi_i)}$ and the signal-to-noise ratio (SNR) values can be computed as

$$\mathrm{SNR}_{X_i} = \frac{P_{(X_i-\xi_i)}}{P_{\xi_i}}.$$

The initial phase $\omega_i = \omega_i(\nu)$ of each channel's driving signal is sampled from an independent uniform distribution on period $[0, 2\pi)$. A signal's power and its SNR are positive real-valued random variables, depending on ω_i in general.

The signal recorded by channel 1 is defined by:

$$\begin{cases} I_1(t,\nu) &= 2\,\mathrm{asin}\big(\sin\big(2\pi f_1 t + \omega_1(\nu)\big)\big); \\ X_1(t,\nu) &= I_1(t,\nu) + \xi_1(t,\nu). \end{cases} \tag{1}$$

The average power of channel 1 is $P_{(X_1-\xi_1)} = P(2\,\mathrm{asin}\big(\sin\big(2\pi f_1 t + \omega_1\big)\big)$, which yields $P_{(X_1-\xi_1)} = \frac{\pi^2}{3}$ for any $f_1 > 0$ and $\omega_1 \in [0, 2\pi)$. Hence,

$$\mathrm{SNR}_{X_1} = \frac{\pi^2}{3}. \tag{2}$$

The signal recorded by channel 2 is defined by:

$$\begin{cases} I_2(t,\nu) &= \begin{cases} 1 & 0 \le (2\pi f_2 t + \omega_2(\nu)) \bmod 2\pi < \pi; \\ -1 & \text{otherwise} \end{cases} \\ X_2(t,\nu) &= I_2(t,\nu) + \xi_2(t,\nu). \end{cases} \tag{3}$$

The average power of channel 2 is $P_{(X_2-\xi_2)} = 1$, because $I_2(t,\nu)^2 = 1$ for any $f_2 > 0$ and $\omega_2 \in [0, 2\pi)$. Hence,

$$\mathrm{SNR}_{X_2} = 1. \tag{4}$$

The signal recorded by channel 3 is defined by:

$$\begin{cases} I_3(t,\nu) &= \cos\big(2\pi f_3 x + \omega_3(\nu)\big) \\ X_3(t,\nu) &= W_{(1,2)} X_1(t,\nu) X_2(t,\nu) + I_3(t,\nu) + \xi_3(t,\nu). \end{cases} \tag{5}$$

The average power of channel 3 is computed as follows

$$\begin{aligned} P_{(X_3-\xi_3)} &= P_{(W_{(1,2)} X_1 X_2 + I_3)} \\ &= W_{(1,2)}^2 P_{X_1 X_2} + 2 W_{(1,2)} \varepsilon(\nu) + P_{I_3}, \end{aligned} \tag{6}$$

where

$$\varepsilon(\nu) = \lim_{T\to\infty} \frac{1}{T} \int_{-T/2}^{T/2} I_1(t,\nu) I_2(t,\nu) I_3(t,\nu) dt.$$

Given that $X_1^2 = I_1^2 + 2I_1\xi_1 + \xi_1^2$ and $X_2^2 = I_2^2 + 2I_2\xi_2 + \xi_2^2$, the average power of X_1X_2 is

$$P_{X_1X_2} = \lim_{T\to\infty} \frac{1}{T}\int_{-T/2}^{T/2} (I_1^2 + 2I_1\xi_1 + \xi_1^2)\ (I_2^2 + 2I_2\xi_2 + \xi_2^2)dt. \tag{7}$$

Because $|I_2| = 1$, we have

$$P_{X_1X_2} = \lim_{T\to\infty} \frac{1}{T}\int_{-T/2}^{T/2} (I_1^2 + 2I_1\xi_1 + \xi_1^2)\ (1 + 2I_2\xi_2 + \xi_2^2)dt \tag{8}$$

$$= \lim_{T\to\infty} \frac{1}{T}\int_{-T/2}^{T/2} (I_1^2 + 2I_1^2I_2\xi_2 + I_1^2\xi_2^2 + 2I_1\xi_1 + 4I_1I_2\xi_1\xi_2 + 2I_1\xi_1\xi_2^2 + \xi_1^2 + 2I_2\xi_1^2\xi_2 + \xi_1^2\xi_2^2)dt. \tag{9}$$

The white noise signals $\xi_i(t)$ follow a standard Gaussian distribution (mean $\mu = 0$, standard deviation $\sigma = 1$), such that

$$\lim_{T\to\infty} \frac{1}{T}\int_{-T/2}^{T/2} I_1^2I_2\xi_2 dt = 0;$$

$$\lim_{T\to\infty} \frac{1}{T}\int_{-T/2}^{T/2} I_1\xi_1 dt = 0;$$

$$\lim_{T\to\infty} \frac{1}{T}\int_{-T/2}^{T/2} I_1I_2\xi_1\xi_2 dt = 0;$$

$$\lim_{T\to\infty} \frac{1}{T}\int_{-T/2}^{T/2} I_1\xi_1\xi_2^2 dt = 0;$$

$$\lim_{T\to\infty} \frac{1}{T}\int_{-T/2}^{T/2} I_2\xi_1^2\xi_2 dt = 0;$$

Then, equation (9) can be rewritten as

$$P_{X_1X_2} = P_{I_1} + 0 + \lim_{T\to\infty} \frac{1}{T}\int_{-T/2}^{T/2} I_1^2\xi_2^2 dt + 0 + 0 + 0 + \lim_{T\to\infty} \frac{1}{T}\int_{-T/2}^{T/2} \xi_1^2 dt + 0 + \lim_{T\to\infty} \frac{1}{T}\int_{-T/2}^{T/2} \xi_1^2\xi_2^2 dt \tag{10}$$

$$= P_{I_1} + P_{I_1\xi_2} + P_{\xi_1} + P_{\xi_1\xi_2}. \tag{11}$$

Notice that $P_{I_1} = \frac{\pi^2}{3}$ according to equation (1) and $P_{I_1\xi_2} = \frac{\pi^2}{3}$ because I_1^2 is independent of ξ_2^2 and $P_{\xi_1} = 1$ by definition and $P_{\xi_1 xi_2} = 1$ because ξ_1^2 and ξ_2^2 are mutually independent. Hence, we obtain

$$P_{X_1X_2} = \frac{\pi^2}{3} + \frac{\pi^2}{3} + 1 + 1$$

$$P_{X_1X_2} = \frac{2\pi^2}{3} + 2. \tag{12}$$

For each $f_3 > 0$, $\varepsilon(\nu) = 0$ with probability 1 as $\omega_i(\nu)$ is uniformly distributed in $[0, 2\pi)$. According to equation (5), $P_{I_3} = \frac{1}{2}$. Hence, equation (6) can be rewritten as

$$P_{(X_3,\xi_3)} = W_{(1,2)}^2 P_{X_1X_2} + 2W_{(1,2)}\varepsilon(\nu) + P_{I_3}$$

$$= W_{(1,2)}^2(\frac{2\pi^2}{3} + 2) + 0 + \frac{1}{2}, \tag{13}$$

which leads to the signal-to-noise ratio for channel 3 equal to

$$\mathrm{SNR}_{X_3} = \left(\frac{2}{3}\pi^2 + 2\right) W_{(1,2)}^2 + \frac{1}{2}. \tag{14}$$

S2. A simpler three-channel model of bifrequencies interaction

The main body of the paper describes a model with three channels that incorporates both triangular and rectangular waveforms. This design aims to achieve a complex power distribution in the model, resembling the patterns commonly observed in natural phenomena. Both triangular and rectangular waveforms consist exclusively of odd harmonics. Specifically, rectangular waves are characterized by odd harmonics of the form $2k + 1$, where the amplitude is proportional to to $1/(2k + 1)$. Triangular waves also consist of odd harmonics, but their amplitude is proportional to $1/(2k + 1)^2$.

A simplified three-channel model of bifrequency interactions, represented by sinusoidal waves $I'_1(t, \nu)$ and $I'_2(t, \nu)$, possesses the same power as the functions $I_1(t, \nu)$ and $I_2(t, \nu)$ used in the main body of the paper. This model maintains the same signal-to-noise ratio (SNR) for each channel and enables investigation into the extent to which the harmonics of the carrier waves in the input channels can affect the detection of quadratic phase coupling, according to the metrics presented in the main text. Therefore, the simplified three-channel model of bifrequency interactions is defined by the following functions:

$$I'_1(t, \nu) = \frac{\sqrt{6}\pi}{3} \sin\big(2\pi F_1 t + \omega_1(\nu)\big); \tag{15}$$

$$I'_2(t, \nu) = \sqrt{2} \sin\big(2\pi F_2 t + \omega_2(\nu)\big); \tag{16}$$

$$I'_3(t, \nu) = I_3(t, \nu) = \cos\big(2\pi F_3 t + \omega_3(\nu)\big). \tag{17}$$

We have conducted an experiment identical to that described in the main text, by merging 128 independent epochs, with each epoch corresponding to a 5-second interval sampled at 100 Hz. This was tested under the same conditions, using the same set of frequencies and the same coupling strengths $W_{(1,2)}$ between interacting channels X'_1 and X'_2. Following the procedure of the main study, for all 354 experimental conditions, we computed the R_{BQPC} (i.e., the ratio of bispectral quadratic phase coupling) and the R_{GQPC} (i.e., the ratio of bivariate Granger causality quadratic phase coupling).

We observed that the coupling strength $W_{(1,2)}$ influenced the metrics used to detect QPC (Figure S1, Figure S2). The results were very similar to those obtained using the original triangular and rectangular periodic functions. This comparison is evident when contrasting Figure 4 of the main text with Figure S1 in the supplementary materials. Moreover, even with the oversimplified model signals, the impact of the coupling strength between the interacting channels mirrors the observations made with the original signals discussed in the main paper. We noted a similar discrepancy between the experimental and analytical values of signal-to-noise ratios in channel X'_3. This discrepancy became more pronounced at lower values of $W_{(1,2)}$, accompanied by a broader dispersion in the ratio between experimental and corresponding analytical values, as seen when comparing Figure S2A with Figure 5A in the main text. Additionally, with the simplified model signals, the bispectral method proved to be superior to bivariate Granger causality for detecting QPC, as demonstrated by the comparison of Figure S2B to compare with Figure 5B in the main text.

In summary, the results obtained with simplified sinusoidal periodic functions are highly consistent with those obtained using triangular and rectangular waveforms as presented in the main text. This observation indicates that the waveform of the input signals plays a minimal role in the measurement of QPC based on the metrics newly described in the main text. These findings suggest that the assessment of QPC by R_{BQPC} and R_{GQPC} is largely unaffected by the waveforms of the input signals. This could represent a significant advantage for applications in time series analysis obtained from biological contexts, particularly in the study of neural signals.

References

Grami, A. (2015). *Introduction to Digital Communications.* 604 pp. Academic Press, 1st edition.

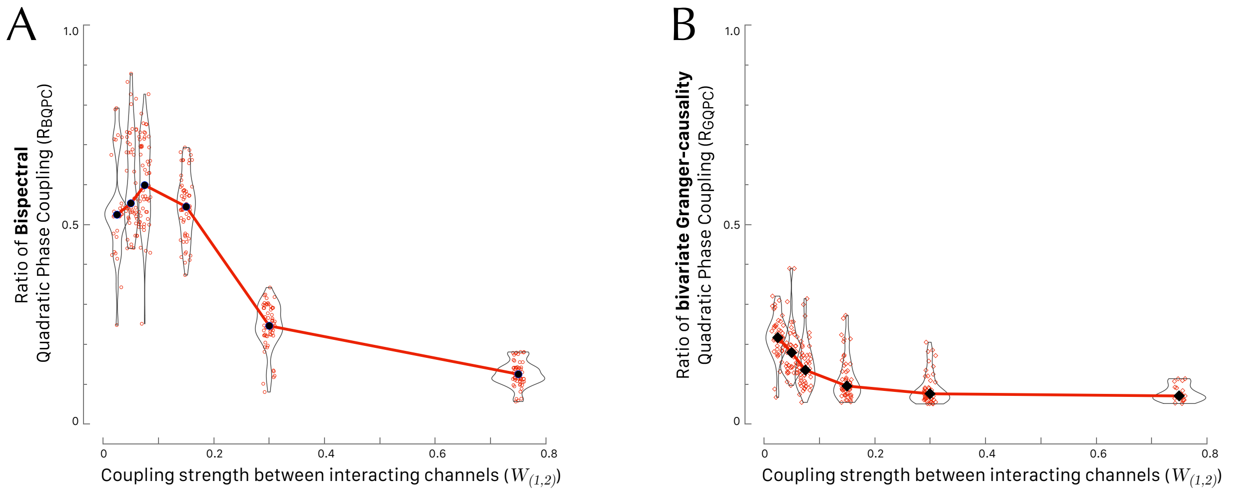

Figure S1: Effect of coupling strength between interacting channels X_1' and X_2' on the method chosen to detect quadratic phase coupling. This figure is similar to Figure 4 of the main text, but the current figure is the result of the analysis of an oversimplified model with only sinusoidal periodic signals. **A**: Ratio of bispectral quadratic phase coupling (R_{BQPC}) as a function of the coupling strength $W_{(1,2)}$ between X_1' and X_2'. **B**: Ratio of bivariate Granger causality Quadratic Phase Coupling (R_{GQPC}) as a function of the coupling strength. For graphical purpose the overlay of points has been limited by adding a random horizontal jitter equal to 0.05.

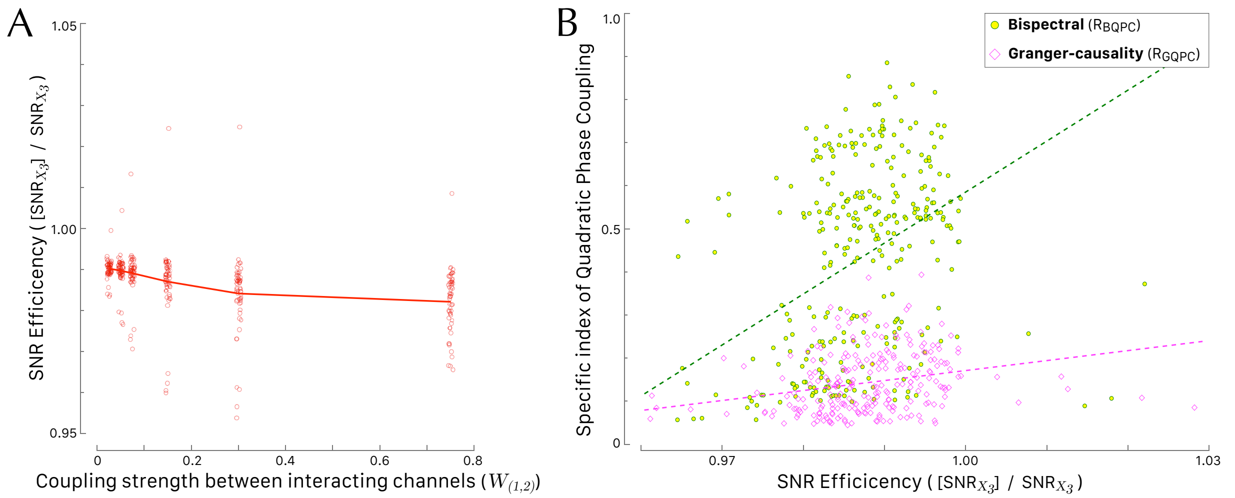

Figure S2: A: Effect of coupling strength between interacting channels X_1 and X_2 on SNR Efficiency of channel X_3. Compare this plot with Figure 5A in the main text. The plot includes the result for all 354 conditions and for graphical purpose the overlay of points has been limited by adding random horizontal and vertical jitters of 0.005 and 0.002, respectively. **B**: Effect of SNR Efficiency observed in channel X_3' with the specific QPC metrics associated with interacting channels X_1' and X_2'. The dashed lines computed according to Pearson's correlation are very similar to the observation made with the original signals, i.e. compare with Figure 5B in the main text. For graphical purpose the overlay of points has been limited by adding a random horizontal and vertical jitter equal to 0.008.
